# Ethnobotanical survey of cosmetic plants used in Marquesas Islands (French Polynesia)

**DOI:** 10.1186/s13002-016-0128-5

**Published:** 2016-11-29

**Authors:** Xénia Jost, Jean-Luc Ansel, Gaël Lecellier, Phila Raharivelomanana, Jean-François Butaud

**Affiliations:** 1 Ecole Nationale Supérieure Agronomique de Montpellier SupAgro, 2 pl. Viala, Montpellier Cedex 02, 34060 France; 2 Cosmetic Valley, 1 pl. de la Cathédrale, Chartres, 28000 France; 3 Ecologie Marine Tropicale des Océans Pacifique et Indien, ENTROPIE UMR 250/9220 IRD-CNRS-UR, 101, promenade Roger-Laroque, Nouméa Cedex, 98848 New Caledonia; 4 University of Paris Saclay - Versailles-Saint Quentin en Yvelines, 55 Avenue de Paris, Versailles, 78035 France; 5 University of French Polynesia, EIO UMR 241, B.P. 6570-98702 Faa’a, Tahiti, French Polynesia; 6 Consultant in forestry and Polynesian botany, B.P. 52832-98716 Pirae, Tahiti, French Polynesia

**Keywords:** Cosmetopoeia, Cosmetics, French Polynesia, Marquesas Islands, Pacific Ocean, Ethnobotanical survey

## Abstract

**Background:**

Cosmetic plants and their uses have often been neglected in ethnobotanical surveys which focus mainly on plants with medicinal or food uses. Thus, this survey was carried out to specifically investigate cosmetics in a small community and to establish a cosmetopoeia, based on the model of pharmacopoeia for medicinal plants. The geographic spread of the survey covered the Marquesas Islands, one of the five archipelagos of French Polynesia (Pacific Ocean). This archipelago was also recently investigated for its pharmacopoeia.

**Methods:**

This survey is based on individual interviews of Marquesan informants on the islands of Tahiti (Society archipelago) and Nuku Hiva (Marquesas archipelago). The methodological approach was semi-directive with open-ended questions based on cosmetic criteria (application area, cosmetic use, plant). Before each interview, researchers and the informant signed a Prior Informed Consent (PIC). Quantitative analyses were performed using basic statistics and the indice of Fidelity Level (FL).

**Results:**

Twenty-eight informants from five of the six inhabited Marquesan islands were interviewed and yielded more than 500 cosmetic recipes. Marquesan cosmetopoeia included 79 plant taxa, of which 5% are Marquesan endemics, 23% are indigenous, 28% are Polynesian introductions and 44% are modern introductions. Among the introduced species, half were cultivated whereas the other half were weedy species. Most of the plants were abundant and only eight species were considered rare, of which four were Marquesan endemics. Main cosmetic plants were identified through informant citations and fidelity levels, and included *Calophyllum inophyllum*, *Cananga odorata*, *Citrus aurantiifolia*, *Cocos nucifera*, *Curcuma longa*, *Gardenia taitensis*, *Mentha* spp., *Ocimum basilicum*, *Rauvolfia nukuhivensis* and *Santalum insulare* var. *marchionense*. The most referred application areas were skin, hair and private parts whereas the main cosmetic uses were perfume, hydration, medicinal care and healing.

**Conclusions:**

Through this survey, Marquesan cosmetopoeia was investigated in detail and uncovered a majority of introduced and abundant plants, and a minority of endemic and rare plants which required proper management to avoid future shortage. The well known perfumed coconut oil or monoi appeared as the main Marquesan cosmetic preparation either for the skin and the hair. Several plants and preparations warrant scientific investigations for their originality.

## Background

**Fig. 1:**
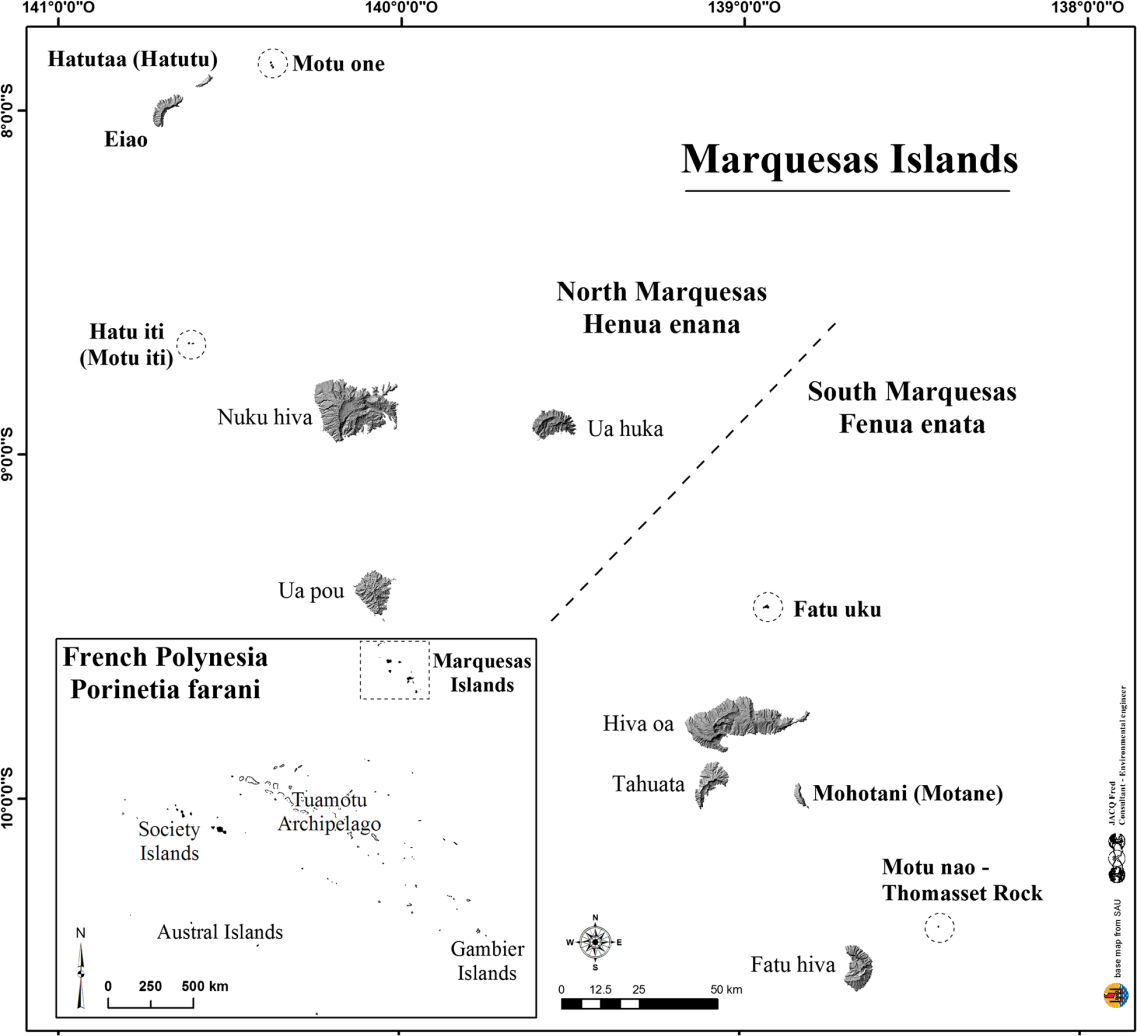
Map of the Marquesas Islands in French Polynesia

French Polynesia belongs to Eastern Polynesia and is located in the centre of the Pacific Ocean, between latitudes 7–28° S and longitudes 134–155° W, with an exclusive economic zone of around 4.8 millions km^2^ and a total land area of 3521 km^2^ [1]. This French overseas country is composed of 118 islands and 5 archipelagos, namely the Society Islands (where the well known islands of Tahiti and Bora Bora are situated), the Austral Islands, the Tuamotu archipelago (only atolls), the Gambier Islands and the Marquesas Islands, for a population of 268,270 inhabitants in 2012 [2]. The Marquesas archipelago, subject of the present survey, is located in the North-East of French Polynesia (Fig. 1): it is the least populated (9264 inhabitants) and the most isolated from continents in the world. With its thirteen islands accounting for a total land area of 1050 km^2^, Marquesas Islands constitute 30% of all Polynesian territory, but only six islands are inhabited with 3.5% of Polynesian population. The Marquesas archipelago is usually divided into the Southern Marquesas Islands including Hiva Oa, Tahuata and Fatu Hiva (3504 inhabitants; 479 km^2^), and the Northern Marquesas Islands with Nuku Hiva, Ua Pou and Ua Huka (5760 inhabitants; 531 km^2^). Located between latitudes 7–10° S, its climate is nearly dry tropical with average annual rainfalls of 1000–1800 mm and an average annual temperature of about 26 °C at sea level [3]. The Marquesan flora is well known with 350 native species including 187 endemic species (53% Marquesan endemism), around 65 species introduced during Polynesian migrations more than 1000 years ago (Polynesian introductions) and probably more than 800 species introduced more recently since the end of the 18th century and the European rediscovery of the archipelago (modern introductions) [4–7].

Cosmetopoeia consists of studying the traditional uses of raw materials, such as plants or minerals, for cosmetic purposes, analogous to a pharmacopoeia for medicinal plants [8, 9]. It aims at gathering a better knowledge of this world cultural heritage, preserving those uses and promoting the biosourcing potential of natural ingredients for body care products. Traditional uses of cosmetic plants belong and are specific to each community from whom they originated, and particularly for the Polynesians inhabiting Pacific Ocean islands. Indeed, French Polynesia and particularly the Marquesas Islands [10], which possess a strong cultural identity [11], is well known throughout the world for perfumed coconut oil called monoi [12]. Natural fragrance is an important aspect of Marquesan beauty standards. Fragrant flowers are also omnipresent in traditional cosmetic preparations (*mono’i* in Tahitian or *pani* in Marquesan) and can be directly applied to the hair or the skin.

The implementation of ethnobotanical studies dedicated to cosmetic plants is necessary due to their underrepresentation in such studies and the lack of details on their preparations and their uses [9, 10, 12–15]. Thus, the present survey reports the first field investigation of French Polynesian cosmetopoeia and focuses on traditional Marquesan plants used in cosmetics. It follows up an ethnobotanical investigation of Marquesan pharmacopoeia that was conducted previously by Girardi et al. [16].

## Methods

### Geographic coverage

Ethnobotanical surveys were performed on Tahiti (Society Islands) and Nuku Hiva (main island of the Marquesas archipelago) with Marquesan traditional practitioners from Nuku Hiva, Ua Pou and Ua Huka in the Northern Marquesas, and Tahuata and Fatu Hiva in the Southern Marquesas (Table 1).

**Table 1:** Informants data and island of origin

Marquesas	Island	Number of informants	Female proportion of informants	Inhabitants	Female proportion of inhabitants
North	Nuku Hiva	17	76%	2966	47%
North	Ua Pou	2	50%	2173	50%
North	Ua Huka	1	100%	621	48%
North	Total 3 islands	20 (71%)	75%	5760 (62%)	48%
South	Hiva Oa	0	-	2190	47%
South	Tahuata	2	100%	703	49%
South	Fatu Hiva	6	100%	611	47%
South	Total 3 islands	8 (29%)	100%	3504 (38%)	47%
Marquesas	Total 6 islands	28	82%	9264	48%

### Ethnobotanical data collection

Informants were interviewed in Tahiti in May–July 2014 and June 2015, and in Nuku Hiva between 5–18 August 2014. In Tahiti, we interviewed them in their homes, as numerous Marquesans are living in Tahiti, and in an annual handicraft exhibition, gathering craftsmen from most of the Marquesas Islands come to the town of Pirae. In Nuku Hiva, we led our interviews in their homes as well, in all the villages of the island: Taiohae, the main village, Taipivai, Hooumi, Hatiheu and Aakapa. Regarding the investigations in Tahiti, we kept only coherent Marquesan preparations free of Tahitian influences (only Marquesan plant names, plants found in Marquesas Islands). All informants were selected and consulted for their rich knowledge of plants and traditional uses but also because they still continue to formulate cosmetic preparations for their friends, family or for tourists. We also focused on informants interviewed by Girardi et al. [16] for the Marquesan pharmacopoeia and on the informants recommended by members of “*Académie marquisienne*” or Marquesan Academy, specialists in Marquesan language and culture.

Before starting the interview, the interviewers had to introduce themselves and explain clearly to the informant the aims of the study. Prior to each interview process, a free informed consent form (PIC, Prior Informed Consent) had to be signed by both interviewer and informant (topic, objectives, goals of the study, respect of confidentiality); this PIC dealt also with access and benefit-sharing (ABS) agreements with the commitment taken by the researchers to associate individual informants, or a representative entity grouping them, to eventual assertion of Intellectual Property rights (patent) and commercial applications from cosmetic plant biochemical studies. The majority of interviews were individual, sometimes with two or three persons if translation was required when the informant spoke only in Marquesan language. There were no refusals to participate, to sign the PIC form or to answer our questions.

The methodological approach was semi-directive with open-ended questions. The proposed method offered a list of allegations per target or per use. The survey was based on three standards: application area (hair, body, face) with practices specific to each area of application, uses (care, protection, hygiene, embellishment and perfume) with allegation lists linked to each application area, and ethnobotany. The interviewer chose an application area, and then asked open-ended questions per target (for example, if “body” as area, then skin, stomach, breasts, etc. as target). The interviews sought to determine the type of plants used, purposes of utilization, parts used, treated problems, modes of preparation and method of administration. An allegation is a use for a specific target, for example: anti-dandruff care for hair. If two different persons quote an identical allegation, we counted two allegation reports. Likewise, if two different preparations indicated an identical allegation, we counted two allegation reports. As the investigation progressed, all allegations proposed in the list were compiled and analysed. We took into account all the data given by informants, even including some allegations not present in the cosmetic list: these allegations were usually considered as “pharmacopoeia allegations”. Therefore, one preparation can present several application areas, several targets, and/or several allegations.

### Plant identification

The identification of each plant used for cosmetic treatments (quoted by its Marquesan name or showed to the interviewer) was facilitated by previous work done on Marquesan pharmacopoeia with numerous informants in common in both studies and with voucher specimens deposited at PAP herbarium in Tahiti [16], by botanical booklets aiming at linking Latin and Marquesan plant names [5, 6] and by the last author, botanist, who checked plant names and pictures reported by the interviewers. Several species of mints and frangipanis were clustered into unique taxa (ethnospecies), respectively *Mentha* spp. and *Plumeria* spp., because these plants are morphologically very close and are equally used for the same cosmetic uses in similar recipes.

### Data analysis

The concept of an “ethnobotanical event” adapted from Tardio et al. [17] by Girardi et al. [16] for the study of Marquesan herbal medicine was implemented with the definition of allegation reports. A table gathering all data obtained from the investigations (informant, recipe, species, plant part, target, cosmetic use, allegation) was built to compile in different lines each allegation report so that each parameter could be analyzed independently. Another table was built with informant features: name, age, date and place of interview, number of recipes, occupation. Before initiating any analysis, grouping of allegations and various parts of plants were done in the first completed data table in order to homogenize all data; for example: treat injuries/heal wounds/tend scratches = healing, or grain/pod/fruit = seed. From this network, other tables and graphs were realised as well as statistical analysis with XLSTAT software (2014.4.02).

One of the most popular indexes in ethnopharmacology is the fidelity level (FL) [18, 19] and is implemented to quantify, for a group of people, the importance of one plant for a specific treatment. This index is calculated using the following formula FL (%) = 100 NIUE/NIU, where NIUE is the number of informants quoting one plant species for a particular use, and NIE is the number of informants quoting that plant for any use. The more the value of FL is close to 1, the higher is the number of informants that used this plant species for that particular use. This index answers the question: “Which use is associated to this particular plant?”

Concerning the informants data, a correlation with a scatter plot in XLSTAT has been tested between parameters “number of recipes” and “number of allegations”. A two-dimensional graph was built: the vertical axis represents the “number of recipes” value, and the horizontal axis represents the “number of allegations” value.

## Results and Discussion

### Informants and geographical coverage

A total of 28 Marquesan informants were interviewed, 23 women (82%) and 5 men (18%), ranging from 33 to 84 years old (Table 1). More women were interviewed than men, but it was not intentional: informants possessing a high knowledge about plants and their uses were targeted and were selected by Marquesan resource persons (Marquesan Academy or other informants). It is a fact that essentially women prepare and use cosmetic recipes, except for tattoo and that their traditional cosmetic knowledge was inherited from their mother or their grandmother, mostly from female lineage. Moreover, from the interviews, body care seems to be fundamentally a concern among women either for the well being of their children or themselves.

Relative to the geographical coverage, aiming to establish comparisons, we tried to balance interviews according to demographic distribution between Northern and Southern Marquesas, as these two sub-archipelagos are generally distinguished for language and cultural differences [20]. This intention was nearly achieved with 71% (56–86% with a confidence interval of 95%) of the informants coming from Northern Marquesas, which represents 62% of the whole archipelago population (Table 1).

Overview on the knowledge of the 28 informants is given in Table 2 with their geographical origin, and their number of recipes, plant species used and allegation reports. For example, informant 29 came from the Northern Marquesas, gave 230 allegations in 24 different recipes using 28 distinct plant species. Thus, the higher the number of recipes/allegations is, the richer the informant knowledge is. This also provides an idea of the diversity of used plants in the 527 collected recipes.

**Table 2:** Geographical origin (in bracket, number of informants), number of recipes, number of plants and number of allegations per informant (M: male, others: female)

Marquesas	Informant number	Number of recipes	Number of plants	Number of allegations
North	5	17	21	94
	12 (M)	6	5	9
	13 (M)	9	9	42
	16	22	24	117
	17	10	16	97
	18	15	21	75
	19	17	16	84
	20	15	16	73
	22	16	24	129
	23 (M)	26	25	77
	24	17	30	156
	25	10	8	63
	26	25	21	190
	27 (M)	15	13	59
	28	19	16	81
	29	24	28	230
	30	29	24	204
	31 (M)	20	13	111
	32	28	27	171
	33	20	25	118
North (20)	Maximum	29	30	230
	Average	18.0	19.1	109.0
	Minimum	6	5	9
South	3	25	24	59
	6	17	25	198
	7	16	20	112
	9	17	19	87
	10	26	21	127
	21	18	24	172
	35	23	24	171
	36	25	22	124
South (8)	Maximum	26	25	198
	Average	20.9	22.4	131.3
	Minimum	16	19	59
Marquesas (28)	Maximum	29	30	230
	Average	18.8	20.0	115.4
	Minimum	6	5	9
Female (23)	Maximum	29	30	230
	Average	19.6	21.6	127.5
	Minimum	10	8	59
Male (5)	Maximum	26	25	111
	Average	15.2	13.0	59.6
	Minimum	6	5	9

On average, each informant gave 19 recipes (between 6 and 29), involving 20 plant species (between 5 and 30), and resulting in 115 allegation reports (between 9 and 230). No significant differences of knowledge between Northern and Southern Marquesas can be put in evidence, especially if Northern informants 12 and 13, who were males interviewed without the special recommendations of Marquesan Academy, are put aside. If the variations are not geographical, it appears that they are clearly linked with the gender, men having a quarter of recipes less, using 40% less plants and resulting in half the number of allegations in comparison with women (Table 2). This explains why the number of male informants is low: knowledge on cosmetic plants is fundamentally shared among Marquesan women today, even if some men (number 23 educated by its mother and number 31 the elder—84 years old), constituting the exceptions, have a rather similar knowledge.

### Taxonomy of cosmetic plants

According to the interviews, 79 taxa (including species, subspecies and varieties) of cosmetic plants were documented for uses involving different plant parts; for convenience, the word species will be used instead of taxa or taxon in the text henceforth. Scientific name, botanical family name, biogeographical status, names given in Northern or Southern Marquesas, number of informants using them and other botanical data were recorded for each cosmetic plant species (Table 3). These species belong to 47 families, the most represented being Apocynaceae (4), Euphorbiaceae (4), Lamiaceae (4), Poaceae (4), Rutaceae (4), Convolvulaceae (3), Fabaceae (3), Malvaceae (3), Rubiaceae (3) and Zingiberaceae (3). Among the genera, *Citrus* (4), *Gardenia* (2), *Ipomoea* (2) and *Zingiber* (2) encompass several cosmetic species; the genera *Fagraea*, *Mentha* and *Plumeria* could also be mentioned as they comprised several very similar species or cultivars. It is interesting to observe that among the seven tropical tree family quoted by Ansel et al. [8] as being the more quoted in the literature for cosmetic uses in the world, four are also the main ones in the Marquesas, namely Fabaceae, Malvaceae, Rubiaceae and Rutaceae. At a lower level, genera *Gardenia* and *Morinda* (Rubiaceae), *Citrus* (Rutaceae), *Hibiscus* (Malvaceae), *Cocos* (Arecaceae), *Calophyllum* (Calophyllaceae) and *Artocarpus* and *Ficus* (Moraceae) appear in both studies. This exhibits the congruence of cosmetopoeia of the Marquesas Islands and at least the Asia-Pacific region.

**Table 3:** Cosmetic plants cited by informants with botanical information

Family	Scientific name	N/S (1)	Biogeographical status (2)	Cultivation	Abundance (3)	Naturalization status (4)	Type of plant	Local name (N) (1)	Local name (S) (1)	Number of informants
Amaranthaceae	*Achyranthes aspera* L. var*. aspera*	N	Pol	No	3	Nat	Shrublet	mokio	-	2
Asteraceae	*Ageratum conyzoides* L.	N/S	Mod	No	1	Nat	Herbaceous	mei’e, mei’e rore	mei’e, putara	6
Euphorbiaceae	*Aleurites moluccanus* (L.) Willd.	N/S	Pol	No	3	Nat	Tree	‘ama	‘ama, ti‘a’iri	5
Xanthorrhoeaceae	*Aloe vera* (L.) Burm.f.	N/S	Mod	Yes	2	Cult	Herbaceous	-	-	1
Apocynaceae	*Alyxia stellata* var*. marquesensis* (F.Br.) Fosberg & Sachet	N	End	No	5	-	Shrub	mei’e papa	-	5
Amaranthaceae	*Amaranthus viridis* L.	S	Pol	No	1	Nat	Herbaceous	-	upo‘oti’i	2
Bromeliaceae	*Ananas comosus* (L.) Merr.	N/S	Mod	Yes	1	Subsp	Herbaceous	-	-	3
Apiaceae	*Anethum graveolens* L.	N/S	Mod	Yes	2	Cult	Herbaceous	taretare	-	1
Marratiaceae	*Angiopteris evecta* (J.G. Forst.) Hoffm.	N/S	Ind	No	3	-	Fern	pa’ahei, puhei	pa’ahei	2
Annonaceae	*Annona muricata* L.	N/S	Mod	Yes	1	Subsp	Tree	-	-	4
Moraceae	*Artocarpus altilis* (Parkinson ex Z) Fosberg	N/S	Pol	Yes	1	Subsp	Tree	‘uru, maiore	‘uru	3
Plantaginaceae	*Bacopa monnieri* (L.) Wettst.	N	Ind	No	3	-	Herbaceous	heiotona	-	1
Lecythidaceae	*Barringtonia asiatica* (L.) Kurz	N	Ind	No	3	-	Tree	hutu	-	1
Bixaceae	*Bixa orellana* L.	N/S	Mod	Yes	3	Subsp	Shrub	roku, peni	roku, peni	1
Calophyllaceae	*Calophyllum inophyllum* L.	N/S	Pol	No	2	Nat	Tree	temanu	tamanu, temanu	10
Annonaceae	*Cananga odorata* (Lam.) Hook.f. & Thomson	N/S	Mod	Yes	1	Nat	Tree	moto’i	moto’i	4
Sapindaceae	*Cardiospermum halicacabum* L.	N	Pol	No	3	Adv	Vine	komoka	-	2
Caricaceae	*Carica papaya* L.	N/S	Mod	Yes	1	Nat	Shrub	-	-	1
Poaceae	*Centotheca lappacea* (L.) Desv.	S	Pol	No	3	Nat	Herbaceous	-	‘ohe’ohe	1
Apocynaceae	*Cerbera manghas* L.	N	Ind	No	3	-	Tree	‘eva	-	1
Poaceae	*Chrysopogon zizanioides* (L.) Roberty	N/S	Mod	Yes	2	Cult	Herbaceous	metie, mouku	mouku	8
Rutaceae	*Citrus aurantiifolia* (Christm. & Panz.) Swingle	N/S	Mod	Yes	1	Subsp	Shrub	hitoro	-	10
Rutaceae	*Citrus hystrix* DC.	N/S	Mod	Yes	3	Cult	Shrub	-	remene	5
Rutaceae	*Citrus maxima* (Burm.) Merr.	N	Mod	Yes	2	Cult	Shrub	-	-	2
Rutaceae	*Citrus x sinensis* (L.) Osbeck	N/S	Mod	Yes	3	Cult	Tree	anani	-	2
Arecaceae	*Cocos nucifera* L.	N/S	Pol	Yes	1	Nat	Tree	‘ehi	e’ehi	12
Rhamnaceae	*Colubrina asiatica* (L.) Brongn. var*. asiatica*	N/S	Ind	No	3	-	Vine	tutu	tutu	4
Commelinaceae	*Commelina diffusa* Burm.f.	N	Mod	No	1	Nat	Herbaceous	heiotona	-	1
Cordiaceae	*Cordia subcordata* Lam.	N/S	Ind	Yes	2	-	Tree	tou	tou	1
Asparagaceae	*Cordyline fruticosa* (L.) A. Chev.	N/S	Pol	Yes	1	Nat	Shrub	‘auti	‘auti	2
Cucurbitaceae	*Cucumis anguria* L.	N	Mod	No	4	Nat	Vine	kokopa kira	-	1
Zingiberaceae	*Curcuma longa* L.	N/S	Pol	Yes	2	Subsp	Herbaceous	‘eka	‘ena, re’a	6
Cyperaceae	*Cyperus mindorensis* (Steud.) Huygh	N	Pol	No	1	Nat	Herbaceous	punie poko tava‘i’e, mutie	-	2
Fabaceae	*Erythrina variegata* L.	N	Pol	No	3	Nat	Tree	kenae	-	2
Euphorbiaceae	*Euphorbia hirta* L.	N	Mod	No	1	Nat	Herbaceous	heeheeamata, pokea, eaeamata	-	7
Euphorbiaceae	*Euphorbia sachetiana* (J. Florence) Govaerts	N/S	End	No	5	-	Shrublet	-	-	1
Gentianaceae	*Fagraea berteroana* A. Gray ex Benth. (syn. *F. berteroana* var*. marquisensis* Fosberg & Sachet) native form	N/S	Ind	No	4	-	Tree	pua ’enana	pua, pua ho’ovai ’enata	2
Gentianaceae	*Fagraea berteroana* A. Gray ex Benth. (syn. *F. longituba* M.L. Grant) introduced form	S	Mod	Yes	2	Cult	Tree	-	pua ho’ovai vaikeka’a	1
Moraceae	*Ficus prolixa* G. Forst. var*. prolixa*	N/S	Ind	No	3	-	Tree	aoa	aoa	2
Rubiaceae	*Gardenia jasminoides* Ellis	N	Mod	Yes	2	Cult	Shrub	taina	-	2
Rubiaceae	*Gardenia taitensis* DC.	N/S	Pol	Yes	1	Cult	Shrub	Tia’e, tiare	tiare	10
Malvaceae	*Gossypium hirsutum* var*. taitense* (Parl.) Roberty	N	Ind	No	4	-	Shrub	haha’avai	-	2
Malvaceae	*Hibiscus rosa-sinensis* L.	S	Pol	Yes	1	Subsp	Shrub	-	‘oute pupu	3
Convolvulaceae	*Ipomoea batatas* (L.) Lam.	N/S	Pol	Yes	1	Cult	Vine	-	‘uma’a	2
Convolvulaceae	*Ipomoea pes-caprae* subsp*. brasiliensis* (L.) Ooststr.	N	Ind	No	3	-	Vine	pohue tatahi	-	1
Oleaceae	*Jasminum grandiflorum* L.	N/S	Mod	Yes	1	Cult	Vine	pitate	pitate	6
Fabaceae	*Leucaena leucocephala* (Lam.) de Wit	N	Mod	No	1	Nat	Shrub	-	-	1
Euphorbiaceae	*Manihot esculenta* Crantz	N	Mod	Yes	1	Cult	Shrublet	ara	-	6
Lamiaceae	*Mentha* spp*.*	N/S	Mod	Yes	1	Cult	Herbaceous	mati	mati	18
Polypodiaceae	*Microsorum grossum* (Langsd. & Fisch.) S.B. Andrews	N/S	Ind	No	2	-	Fern	papamoko, metuapua’a	papamo’o	7
Rubiaceae	*Morinda citrifolia* L.	N/S	Ind	No	1	-	Shrub	noni	noni, nono	7
Fabaceae	*Mucuna sloanei* Fawc. & Rendle var*. sloanei*	N	Ind	No	3	-	Vine	‘auto’u	papanuiaohe	3
Musaceae	*Musa x paradisiaca* L.	S	Pol	Yes	1	Cult	Herbaceous	-	-	1
Lamiaceae	*Ocimum basilicum* L.	N/S	Mod	Yes	1	Nat	Shrublet	miri, mini, miri keka’a	miri	8
Convolvulaceae	*Operculina brownii* Ooststr.	N	Ind	No	4	-	Vine	piatakiohoau	-	1
Cactaceae	*Opuntia cochenillifera* (L.) Mill.	N	Mod	Yes	2	Cult	Shrub	-	-	1
Pandanaceae	*Pandanus tectorius* Parkinson ex Z var*. tectorius*	N/S	Ind	No	3	-	Tree	hinako	hinano	5
Passifloraceae	*Passiflora foetida* L.	S	Mod	No	2	Nat	Vine	-	pu’u moina	2
Lauraceae	*Persea americana* Mill.	N	Mod	Yes	1	Subsp	Tree	-	-	1
Phyllanthaceae	*Phyllanthus amarus* Schumach. & Thonn.	N/S	Mod	No	1	Nat	Herbaceous	moemoe	‘au’iki, tuitui	2
Solanaceae	*Physalis angulata* L.	S	Pol	No	2	Adv	Herbaceous	-	kariri	2
Lamiaceae	*Plectranthus scutellarioides* (L.) R.Br.	N/S	Mod	Yes	1	Cult	Shrublet	terevete	terevete	1
Apocynaceae	*Plumeria* spp*.*	N/S	Mod	Yes	1	Cult	Shrub	tipanie	tipanie	1
Araliaceae	*Polyscias scutellaria* (Burm.f.) Fosberg	S	Mod	Yes	1	Cult	Shrub	-	kafeie	1
Lamiaceae	*Premna serratifolia* L.	N/S	Ind	Yes	1	-	Shrub	va‘ova’o	‘avaro, va‘ova’o	6
Myrtaceae	*Psidium guajava* L.	N/S	Mod	No	2	Nat	Shrub	tuava	-	2
Apocynaceae	*Rauvolfia nukuhivensis* (Fosberg & Sachet) Lorence & Butaud	N	End	No	5	-	Tree	tu’eiao	-	11
Brassicaceae	*Rorippa sarmentosa* (Sol. ex G. Forst. ex DC.) J.F. Macbr.	N/S	Pol	No	1	Nat	Herbaceous	mahimahi	mahimahi	1
Poaceae	*Saccharum officinarum* L.	N/S	Pol	Yes	2	Cult	Herbaceous	-	-	4
Santalaceae	*Santalum insulare* var*. marchionense* (Skottsb.) Skottsb.	N/S	End	No	4	-	Tree	puahi, ahi	puahi	7
Sapindaceae	*Sapindus saponaria* L.	N/S	Ind	No	2	-	Tree	koku’u	koku’u	3
Asteraceae	*Sigesbeckia orientalis* L.	N/S	Pol	Yes	2	Nat	Herbaceous	niou	niou, riou	2
Anacardiaceae	*Spondias dulcis* Sol. ex Parkinson	N	Mod	Yes	2	Cult	Tree	vi farani	-	1
Myrtaceae	*Syzygium malaccense* (L.) Merr. & L.M. Perry	N/S	Pol	Yes	2	Nat	Tree	‘ahi’a, kehika	Kehi’a, kehi’a ‘enata, kehika	2
Malvaceae	*Thespesia populnea* (L.) Sol. ex Corrêa	N/S	Ind	No	2	-	Tree	miro, mi’o	mi’o	4
Orchidaceae	*Vanilla x tahitensis* J.W. Moore	S	Mod	Yes	2	Subsp	Vine	-	-	1
Poaceae	*Zea mays* L.	N	Mod	Yes	3	Cult	Herbaceous	-	-	1
Zingiberaceae	*Zingiber officinale* Roscoe	S	Mod	Yes	2	Cult	Herbaceous	re’a blanc		1
Zingiberaceae	*Zingiber zerumbet* (L.) Sm.	N/S	Pol	No	3	Nat	Herbaceous	kopuhi, ‘opuhi	‘opuhi	5

(1) N = used in Northern Marquesas; S = used in Southern Marquesas

(2) Pol = Polynesian introduction; Mod = modern introduction; Ind = indigenous species; End = endemic to the Marquesas Islands

(3) 1 = very common; 2 = common; 3 = uncommon; 4 = rare; 5 = very rare

(4) Nat = naturalized species; Adv = adventive, weedy species; Subsp = subspontaneous species; Cult = cultivated species

### Biogeographical status of cosmetic plants

**Fig. 2:**
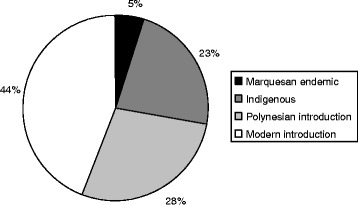
Biogeographical status of cosmetic plants

The flora of Marquesas Islands is usually classified according to the biogeographical status of each plant, between native species which were not introduced by human, Polynesian introductions which were introduced during Polynesian migrations more than one thousand years ago, and modern introductions which were introduced since the end of the 18th century and the arrival of European vessels. Moreover, native species can be divided into endemic species, which are restricted to the Marquesas Islands, and indigenous species, which are also found outside this archipelago. Thus, the majority of cosmetic plants corresponded to modern introductions (44%, 35 species) whereas 28% of them (22 species) were constituted by Polynesian introductions, 23% (18 species) were indigenous, and only 5% (4 species) were endemic to Marquesas archipelago (Fig. 2). This fact showed the importance of introduced species in the present cosmetopoeia and more precisely the value of Polynesian introductions (breadfruit tree, red hibiscus, Tahitian gardenia, cordyline, Malay apple, candlenut tree, shampoo ginger, etc.), which were purposely introduced for their uses and the rapid integration of modern introductions, proof of continuous innovation. The number of native species, accounting for around a quarter of all the cosmetic species, was comparatively low, reflecting perhaps the relative poverty of Marquesan flora compared with other Pacific archipelagos floras [21]. Nevertheless, four endemic plants were parts of these native species and showed the importance of restricted species; however it is worth noting that three of these endemics have close relatives in most of the Pacific in the same species (*Alyxia stellata*) or in the same genus (*Euphorbia*, *Santalum*), indicating the possibility of knowledge transfer through archipelagos.

### Naturalization status of cosmetic plants

**Fig. 3:**
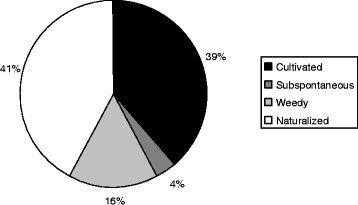
Naturalization status of cosmetic plants

Regarding the naturalization status of the 57 introduced or alien species (Fig. 3), 22 species (39%) are always cultivated, 9 species (16%) are subspontaneous or spreading not far away from former cultivation place, 2 species (4%) are adventive or weeds and 24 species (41%) are naturalized and spreading without any human help. Thus, more than 60% of the introduced species were not cultivated and grew quite freely in disturbed areas like gardens and roads or among natural vegetation. Interestingly, cultivation is not reserved for introduced species which are not naturalized: indeed, two native species (*Cordia subcordata* and *Premna serratifolia*), nine subspontaneous species and seven naturalized species are also cultivated (Table 3); thus, in total, 40 species or half the cosmetic flora, can be obtained by cultivation which enables an easy access to species growing far form the villages or producing not enough materials in the wild. For example, the native shrub *Premna serratifolia* is scattered in dry to mesic forests and is not common enough to allow good harvest of inflorescences; as for the basil *Ocimum basilicum*, it is quite rare at the wild state. The case of the native tree *Fagraea berteroana*, growing in mid to high elevation wet forest, replaced in cosmetic recipes by an introduced cultivar, formerly known under the name *F. longituba*, which is easily cultivated at low elevation in drier places, is symptomatic of the optimization of the cosmetic ingredients harvest (here, the similar flowers of both species).

### Abundance and distribution

Several criteria allowed to characterize plant “abundance” trait: accessibility (mainly distance from houses), and scarcity at the scale of island, valley or island. Precisions on the abundance categories used in Table 3 are given in Table 4. Thus, a cosmetic plant, which is easy to find close to houses, is given the value 1, whereas the one, which is very difficult to find at the scale of the island, is given the highest value 5.

**Table 4:** Definitions of abundance categories

Value	Category	Definition
1	Very common	Easy to find close to all houses
2	Common	Close to houses, but not always all houses
3	Uncommon	Close to few houses, but very scarce in the natural environment
4	Rare	Hard to find in valleys (inhabited area), easier at the scale of the island
5	Very rare	Hard to find at the scale of the valley and the island

**Fig. 4:**
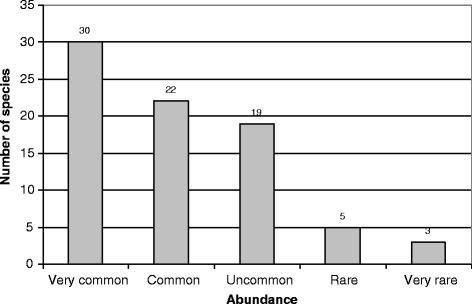
Abundance of cosmetic plants

Two-thirds of Marquesan cosmetic plants are common to very common (Fig. 4), preventing any problem of overexploitation and associated shortage due to their use. Only eight species (10%) are considered rare or very rare: they encompass the four Marquesan endemics plus three indigenous species (*Fagraea berteroana, Operculina brownii*, *Gossypium hirsutum* var. *taitense*) and one modern introduction (*Cucumis anguria*) restricted to dry areas. These seven rare native species are threatened mainly by exotic invasive species which alter their habitats (rats eating their fruits; wild horse, cattle and goats eating their barks, leaves or sprouts; invasive plants choking mother-plants and their regeneration) but some of them are also victims of overexploitation like sandalwood for its fragrant heartwood, or *Rauvolfia nukuhivensis* and *Alyxia stellata* var. *marquesensis* for their barks [22–24].

From a total of 79 plant species (Table 3), 24 species (30%) are exclusively mentioned by people from Northern Marquesas (*Rauvolfia nukuhivensis, Euphorbia hirta, Manihot esculenta, Alyxia stellata,* etc.) and 10 species (13%) by people from Southern Marquesas (*Hibiscus rosa-sinensis, Physalis angulata, Passiflora foetida, Amaranthus viridis*, etc.). Thus, 45 plant species are used for cosmetics in the whole archipelago whereas people from the North seem to possess a wider range of cosmetic species (69 species versus 55 in the South). It is noteworthy to point out that all these exclusive species grow in all the islands of the Marquesas and are mentioned only by one or two informants, except *Rauvolfia nukuhivensis*, which is well known by most of Nuku Hiva community. This species, nearly restricted to Nuku Hiva island in Northern Marquesas (only three living trees known on Ua Huka and probably extinct on Hiva Oa), is frequently used in cosmetics, particularly for intimate hygiene of young girls. The difference between North and South Marquesas could be attributed for the most part to a faint cultural differentiation, as for the Marquesan language [20], but also for a small part to the longer time spent with the Northern Marquesan informants.

### Life forms of cosmetic plants

**Fig. 5:**
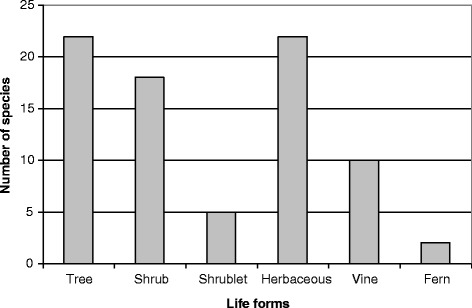
Life forms of cosmetic plants

Among the 79 cosmetic plants, the main life forms (Fig. 5) are trees (coconut tree, screwpine, banyan, sandalwood, Pacific rosewood, etc.) and herbaceous (turmeric, mint, pineapple, sugar cane, etc.) with 22 species for both (28% each), followed by shrubs (Tahitian lime, Tahitian gardenia, frangipani, Polynesian cotton, etc.) with 18 species (23%). Vines (vanilla, sweet potato, jasmine, etc.) are less common with 10 species (13%). Shrublets (basil, tapioca, etc.) and ferns (*Mi* ***c*** *rosorum grossum*, *Angiopteris evecta*) are the least used, with respectively 5 (6%) and two (3%) species. The fern flora, counting for around one third of the native Marquesan species, is then very much the minority among Marquesan cosmetopoeia.

### Plant parts used

**Fig. 6:**
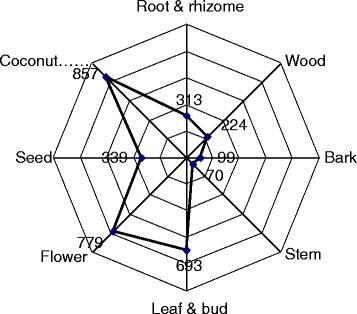
Number of citations per plant parts category

Plants can be used as a whole, especially for small species like *Phyllanthus amarus* or *Cyperus mindorensis*, or for specific parts. An aggregation of plant parts citations was created in order to conduct proper analyses on a few main categories (Fig. 6); for example, the “seed” category includes fruit, nut, kernel, infructescence or pod and the “flower” category includes inflorescence and flower bud. Coconut, which is almost omnipresent, is separated in its own category although it is a nut or seed. Among the different parts of cosmetic plants used by Marquesans, the most reported were the coconut (857 citations—25%), flower (779—23%) and leaf & bud (693—21%). It is remarkable to note the plant parts diversity with all the plant organs used including the wood for the sandalwood, the bark for *Rauvolfia nukuhivensis* or the stem for sugarcane. Moreover, for some plants, several parts are used for cosmetic recipes, like basil (*Ocimum basilicum*), from which flowers, leaves and seeds are used independently.

### Main cosmetic plants

Six plant species are quoted by more than one third of the informants (Table 3): *Mentha* spp. (mints) 64%, *Cocos nucifera* (coconut tree) 43%, *Rauvolfia nukuhivensis* 39%, *Citrus aurantiifolia* (Tahitian lime) 36%, *Gardenia taitensis* (Tahitian gardenia or *tiare*) 36% and *Calophyllum inophyllum* (Alexandrian laurel or *tamanu*) 36%.

**Fig. 7:**
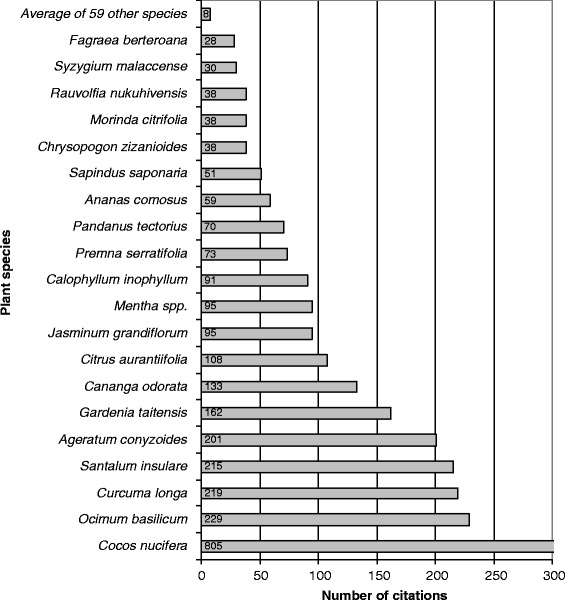
Number of citations per plant species

Regarding the number of citations, *Cocos nucifera* is by far the main species with 805 citations (Fig. 7). This plant is used in many recipes as an excipient; which is an active principle vehicle for a lot of preparations containing several ingredients. Especially in cosmetopoeia, some excipients have cosmetic properties, and could therefore be also considered as active principles. For example, coconut water as well as coconut oil are the main excipients used to prepare cosmetic recipes, but can also be used for their own cosmetic properties. With 229 citations, *Ocimum basilicum* (basil) is the second most important cosmetic plant. The other species with more than 100 citations are *Curcuma longa* (turmeric—219 allegations), *Santalum insulare* var. *marchionense* (Marquesan sandalwood—215 allegations), *Gardenia taitensis* (162 allegations), *Cananga odorata* (ylang-ylang—133 allegations) and *Citrus aurantiifolia* (108 allegations).

These ten species constitute the bulk of the Marquesan cosmetopoeia, which is then composed of two endemic plants, four Polynesian introductions and four modern introductions.

### Cosmetic recipes, allegations and uses

**Fig. 8:**
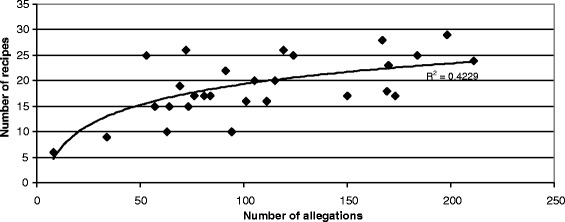
Correlation between number of recipes and number of allegations

The 28 interviews yielded a list of 527 recipes. This study focused on cosmetic uses and a majority of cosmetic allegations were observed. Nevertheless, among these recipes, 6% referred to pharmacopoeia, confirming that there are no clear boundaries in Marquesan culture between concepts of body/aesthetic care and medicinal care, as has been shown by Girardi et al. [16] studying Marquesan pharmacopoeia. Regarding posology, cosmetic recipes could be used every day unlike medicinal recipes. However, recipes addressing skin problems are applied for three days, similar to how a medicine may be applied. Dosages and quantities are not well defined or well measured: plant quantities are measured with handful, hand size or finger size. Adults and children were not subjected to the same dosages to avoid any risk of poisoning as the cosmetic preparations were age and/or weight dose-dependent. As for medicinal recipes [16], each cosmetic recipe contained several plant species (regularly five or more) and can be used on several application areas and for different uses. This leads to a logarithmical correlation between the number of recipes and the number of allegations per informant as shown on the scatter plot of Fig. 8, allegations number increasing exponentially with recipes number. Moreover, each informant knew around 20 cosmetic recipes, which account for around 100 allegations.

**Fig. 9:**
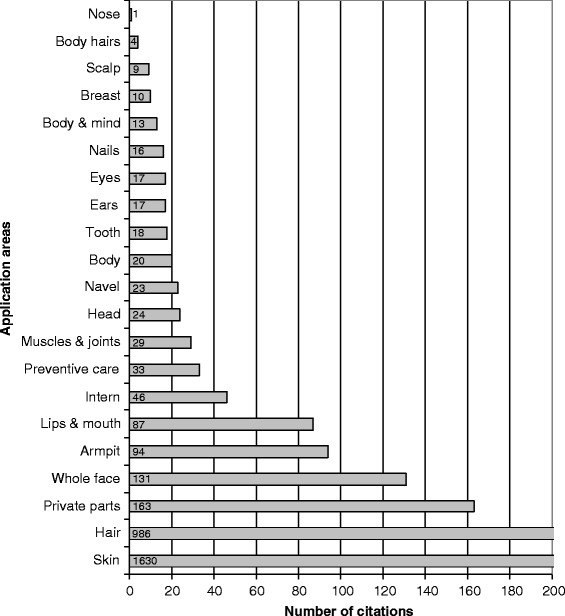
Number of citations per application area

The most referred application areas (Fig. 9) are skin with 1630 allegations (48% of all allegations) and hair with 986 allegations (29%). Private parts come in third position with 163 allegations (5%), followed by whole face (4%), armpit (3%) and lips & mouth (3%) whereas the other 15 application areas account each for less than 2% of all allegations. This analysis indicates key Polynesian cultural traits with the importance of skin and hair care but also a Marquesan originality with private parts as one of the main application areas [12–15].

**Fig. 10:**
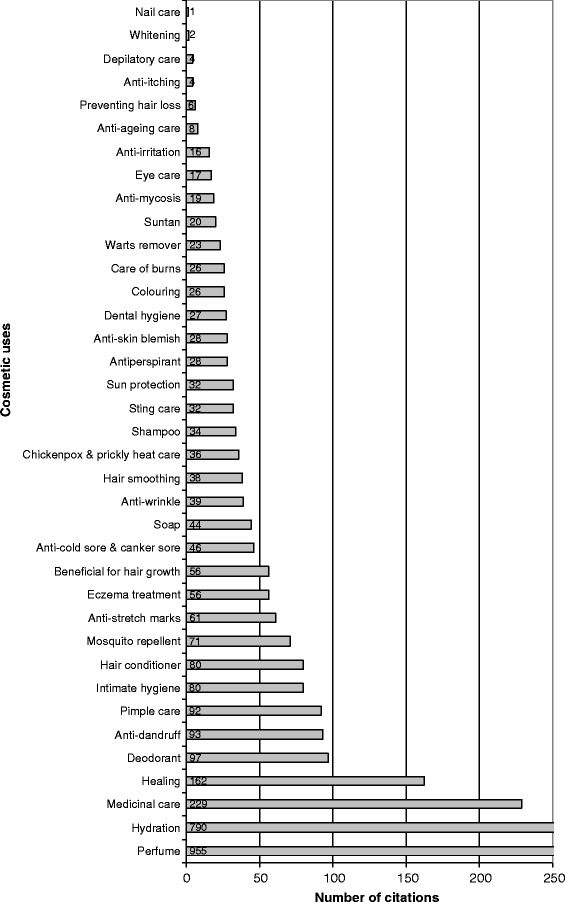
Number of citations per cosmetic use

**Fig. 11:**
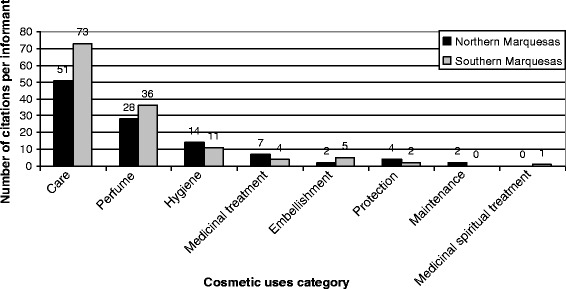
Number of citations per informant for each category of cosmetic uses

**Fig. 12:**
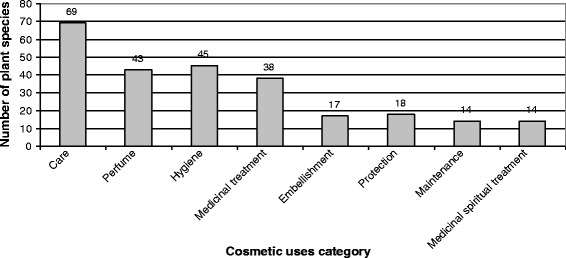
Number of plant species per category of cosmetic uses

The main cosmetic uses can be distinguished from minor ones in Fig. 10 where 37 cosmetic uses have been recognized. Most informants used plants, in cosmetics, for perfume (28% of all allegations), hydration (23%) and healing (5%). Medicinal care was also quoted in 7% of allegations, indicating the close links between Marquesan cosmetopoeia and pharmacopoeia; among these, most visible body care actions could be related to aesthetic and by extension cosmetic concepts. Apart from these four main cosmetic uses, which account for 63% of all allegations, all the other cosmetic uses account for under 3% each of allegations. Marquesan cosmetopoeia appears to be based on the smell (perfume, deodorant, intimate hygiene, soap), on skin care (hydration, medicinal care, healing, pimple care, mosquito repellent, etc.) and on hair care (anti-dandruff, hair conditioner, beneficial for hair growth, hair smoothing, shampoo). A summary of these detailed cosmetic uses in main categories for informants from Northern and Southern Marquesas confirms these characteristics (Fig. 11); moreover, it appears that cosmetic practices from both sub-archipelagos do not differ in terms of cosmetic use categories, both of them indicating care (52%), perfume (27%) and hygiene (10%) as the main categories. Among cosmetic plants, 69 species (87%) are involved in care category whereas 45 species (57%) are used for hygiene and 43 (54%) for perfume (Fig. 12); this indicates utilization of most cosmetic plants in several cosmetic use categories.

### Fidelity level of a species for a particular use

Fidelity levels were calculated for the 575 pairs of cosmetic use - plant species in order to quantify the importance of a species for a particular use. Table 5 indicated the 31 fidelity levels higher than 65% related to use - species associations known by more than one third of the informants. Thus 16 species and seven cosmetic uses appeared to be particularly linked. Fidelity levels were significantly high (several 100% of FL) for several species (*Ageratum conyzoides*, *Cananga odorata*, *Curcuma longa*, *Gardenia taitensis*, *Jasminum grandiflorum*, *Mentha* spp., *Ocimum basilicum*, *Pandanus tectorius* and *Santalum insulare*) used in association with coconut oil for both perfume and hydration, the latter being probably mainly linked with *Cocos nucifera* oil. Two species were closely correlated with female intimate hygiene: the young nut of *Cocos nucifera* and the bark of *Rauvolfia nukuhivensis*, which were also put in evidence in Marquesan pharmacopoeia in preventive care [16]. Healing applications were used mostly with *Calophyllum inophyllum* seed oil, *Cocos nucifera* nut oil or *Santalum insulare* heartwood powder. Finally, the fruits of *Citrus aurantiifolia* and the leaves of *Syzygium malaccense* are commonly used as deodorant and anti-cold sore and canker sore agents, respectively. Among these main fidelity levels, it is striking to note the presence of two Marquesan endemic plants (*Rauvolfia nukuhivensis* and *Santalum insulare* var. *marchionense*) among the six of Marquesan cosmetopoeia; this constitutes real originality and could lead to pertinent phytochemical studies as recently highlighted [25–27]. Regarding coconut oil, its omnipresence is related to its use as an excipient for fragrant preparations but also to its own virtues (active principle) in hydration for example.

**Table 5:** Main fidelity levels for Marquesan cosmetopoeia

Cosmetic use	Plant species	NIUE	NIE	Fidelity level (%)
Hydration	*Ageratum conyzoides*	17	20	85,0
Perfume	*Ageratum conyzoides*	19	20	95,0
Perfume	*Ananas comosus*	11	11	100,0
Healing	*Calophyllum inophyllum*	13	20	65,0
Hydration	*Cananga odorota*	18	20	90,0
Perfume	*Cananga odorota*	20	20	100,0
Deodorant	*Citrus aurantiifolia*	21	23	91,3
Healing	*Cocos nucifera*	23	28	82,1
Hydration	*Cocos nucifera*	27	28	96,4
Intimate hygiene	*Cocos nucifera*	19	28	67,9
Perfume	*Cocos nucifera*	27	28	96,4
Medicinal care	*Cocos nucifera*	20	28	71,4
Hydration	*Curcuma longa*	21	25	84,0
Perfume	*Curcuma longa*	17	25	68,0
Hydration	*Gardenia taitensis*	20	25	80,0
Perfume	*Gardenia taitensis*	19	25	76,0
Hydration	*Jasminum grandiflorum*	10	15	66,7
Perfume	*Jasminum grandiflorum*	14	15	93,3
Hydration	*Mentha* spp*.*	13	16	81,3
Perfume	*Mentha* spp*.*	16	16	100,0
Hydration	*Ocimum basilicum*	18	21	85,7
Perfume	*Ocimum basilicum*	19	21	90,5
Hydration	*Pandanus tectorius* var. *tectorius*	12	13	92,3
Perfume	*Pandanus tectorius* var. *tectorius*	12	13	92,3
Perfume	*Premna serratifolia*	12	16	75,0
Intimate hygiene	*Rauvolfia nukuhivensis*	10	11	90,9
Perfume	*Rauvolfia nukuhivensis*	10	11	90,9
Healing	*Santalum insulare* var*. marchionense*	18	27	66,7
Hydration	*Santalum insulare* var*. marchionense*	25	27	92,6
Perfume	*Santalum insulare* var*. marchionense*	19	27	70,4
Anti-cold sore & canker sore	*Syzygium malaccense*	11	16	68,8

### Description of typical Marquesan cosmetic recipes

Based on traditional Marquesan cosmetic concepts, care is provided through three main recipes categories: monoi, “*paku*” and “*hoho*”.

#### Monoi

Monoi or *mono’i* in Tahitian, which means perfumed coconut oil, is also called *pani* in Marquesan and is known worldwide as a cosmetic oil. Among the three categories, monoi takes the longest time to prepare: the preparation process can take from several days up to a week. Grated coconut, usually from a germinated coconut, is necessarily exposed to the sun in order to obtain the oil, which is later perfumed by the maceration of fresh plant materials for several days. The oil can also be recovered by sun exposure of the coconut milk coming from grated coconut pressing. Macerated plant materials are replaced every two or three days and are used either for their fragrance, like Tahitian gardenia (*Gardenia taitensis*), Spanish jasmine (*Jasminum grandiflorum*), Sweet basil (*Ocimum basilicum*) or for their therapeutic virtues like Alexandrian laurel or *tamanu* in Tahitian/*temanu* in Marquesan (*Calophyllum inophyllum*) or Polynesian sandalwood (*Santalum insulare*). Monoi can be made with only one plant (*pani pitate, pani tia’e, pani temanu…*) or with a combination of several species (*pani kumuhei*). Monoi is used for the softness and the care of body and hair, with fragrance as a key part. These preparations can be applied daily.

#### *Paku*

The term “*paku*” is mainly known in Northern Marquesas but informants from the whole archipelago have recipes that match its description. Two types of *paku* are identified: the first is dedicated to the care and beauty of hair and skin; the second is mostly dedicated to children, even newborns, as a preventive treatment, in order to care cradle cap, to avoid bad smells and to limit vaginal discharges. It has also both medicinal and spiritual uses, the latter being to drive away evil spirits, particularly for babies. *Paku* preparations involve only fresh plants (one or several species), which are often crushed in coconut milk or grated coconut; usually, little (one to two hours) to no sun exposure is needed. This preparation is to be applied immediately by massage of the obtained juice on the targeted part of the body (head, armpit, private parts, all the body) and can be kept on the body for a whole day. *Paku* is not used daily but can be applied until the issue at hand is alleviated. The main plants used for *paku* are *Ageratum conyzoides*, *Cocos nucifera*, *Colubrina asiatica* var. *asiatica*, *Curcuma longa*, *Gossypium hirsutum* var. *taitense* and *Sapindus saponaria*; it should be noted that *Colubrina asiatica* and *Sapindus saponaria* are well known for their uses as soap.

#### *Hoho*

The term “*hoho*” is known only in the island of Fatu Hiva in Southern Marquesas. Only one type of *hoho* exists even if different plants can be used. *Hoho* is mainly aesthetic for the beauty of skin and hair, and for perfume; it complements care afforded by monoi and *paku* and is essentially intended for seduction. *Hoho* requires a longer preparation time than *paku* and can take up to half a day to produce. The preparation involves numerous fresh plants which are crushed in grated coconut; no sun exposure is needed. The preparation is applied on the hair and the body without any filtration and kept for several hours or even the whole day. *Hoho* is not used daily but can be applied at needed. The main plants used for *hoho* are *Ageratum conyzoides, Angiopteris evecta, Cocos nucifera, Curcuma longa, Mentha* spp. and *Ocimum basilicum*.

### Description of particular care types

In order to give an overview of cosmetic uses in a Marquesan life, either daily or more infrequently, several particular care types are described hereunder.

#### Skin and hair

*Paku* and monoi are mostly used for hair and body care (hydration, perfume). Daily massage is very important, providing well-being. It is also used as a cure for some illnesses. For hair care, natural shampoos are mainly made of shampoo ginger (*Zingiber zerumbet*), conditioner made with monoi and anti-dandruff care (*paku* and monoi). There are also specific preparations dedicated to promote hair growth, or to avoid hair loss. For skin problems such as eczema, injuries or fungal infections, Marquesan people used mostly *tamanu* oil (*Calophyllum inophyllum*) for three days or more if the problem persists. In the Marquesas Islands, antiperspirant preparations are often used to avoid perspiration and bad smells. Concerning acne, which is not widespread in Marquesas Islands, only a few preparations were reported. As for depilatory treatments, body hair is naturally scarce in Marquesan population, and is wildly tolerated. Only one person reported a preparation inducing hair loss, composed of leaves, fruits and stems of *Leucaena leucocephala*, which is well known to promote hair loss in the manes of Marquesan horses. Primarily women take care of their body and use in most cases preparations based on natural plants. In some cases, men also groom themselves by applying monoi from time to time on their body; mostly to protect themselves from twigs scratches before a hunting expedition in the bush or from the cold during fishing or navigation. Girls were trained in self-grooming and body care by their mothers, grandmothers or their aunts. For boys, after a certain age, body care is no longer considered important with the exception of frequent bathing.

#### Baby care

Baby care regiments are regularly followed from birth. Daily baths are given with or without boiled leaves from soursop (*Annona muricata*) or *tamanu* (*Calophyllum inophyllum*) for calming effect and skin care. The baby is systematically massaged, during or after the bath, in order to relax him and to ensure good development. Adding some drops of monoi in the bath provides benefits in treating skin problems as: chickenpox, prickly heat or cradle cap. Marquesan people pay attention to the healing of the navel (*pito*) of newborns, especially for boys: to properly dry the umbilical cord and to avoid formation of an outward navel, sandalwood powder (*Santalum insulare*) is applied in a compress attached to the base of umbilical cord, on the navel, for several days.

#### Intimate hygiene

Preparations concerning intimate hygiene are typical of the Marquesas Islands in French Polynesia. Care of private parts is very important for women starting from a very early age. These treatments are preventive actions in order to avoid bad smells and to limit vaginal discharges in adulthood. Three types of preparations were used: the first one is a *paku* composed of a very young green coconut or *koi’e* (*Cocos nucifera*), the second one, restricted to Ua Pou Islands, make use of roots of *Achyranthes aspera*, and the third one is based on a preparation using bark of *tu’eiao* (*Rauvolfia nukuhivensis*), a rare Marquesan endemic tree nearly endemic to Nuku Hiva island, and considered as an endangered species. Preparations are applied every day for several weeks, in small doses, as a few drops in the vagina, for preventive care. Later female personal hygiene consists merely of usual wash and sometimes the use of scented plants in their underwear for fragrance. These intimate care treatments are only for the female gender. Nevertheless, there is one treatment dedicated specifically for men, considered as aesthetical: the supercision.

#### Supercision

This traditional practice existed before the Christian missionary’s arrival. In French Polynesia, and especially in the Marquesas islands, supercision or incision of foreskin (*tehe* in Marquesan) is a masculinity and beauty marker, and is also a hygienic practice to avoid bad odors. The equivalent treatment for female genitalia is the *paku*. A man who is not supercised, is usually badly considered by the community because this practice is considered a culturally significant marker of courage and virility and as a ritual passage from childhood to manhood. This practice is carried out in boys aged from 10 to 15, by a man of the village, and never by a woman. Before the operation, genitals need to be cleaned. A hard coconut shell, or a piece of wood, was placed between the foreskin and the penis of the young boy. The skin was cut using a razor blade, or a piece of sharpened bamboo. Then, the young boy went swimming to the ocean in order to clean and heal the wound, and afterwards, he placed warm pebbles from the beach around his penis to relieve pain and to avoid inflammation. To facilitate healing, monoi is applied every day. Nowadays, circumcision takes place in hospitals and is done by nurses under medical supervision.

#### Tattoo

Tattoo, *tatau* in Tahitian and *patutiki* in Marquesan, can also be considered as an aesthetic practice for both men and women of a certain age, and plants are used to make the ink mixture and to cicatrize the tattooed areas. The tattoo is not only a social status marker but also a sign of beauty and maturity. In the past, to make the ink, nuts of candlenut tree (*Aleurites moluccanus*) were used. Ten nuts were collected and their seeds were threaded on a rib of coconut leave. Then, seeds were sun-dried during two days and ignited in a top-down way, as a candle. A mother of pearl or an empty coconut shell was positioned above this traditional candle in order to recover fumes and soot. Soot was mixed with coconut water (*Cocos nucifera*), which is considered naturally sterile. Ink could also be obtained from charcoal or pounded *Mucuna sloanei* leaves. Tattooing is done using a wooden comb, tortoiseshells, human bones or shark teeth. Monoi may be applied after tattooing to soothe, to prevent dry skin and to help cicatrisation process.

### Evolution of cosmetopoeia through time

The study of cosmetopoeia evolution was made possible only for plants biogeographical status through a literature survey of French Polynesia cosmetic plants [28]. It appears that, nowadays, more introduced plants are used, 72% versus 40% in the past, which can be considered a rapid integration of new plants and an adaptation of cosmetic uses to plants with easy access. Endemic species were more commonly used in the past with 27% of the cosmetic plants for the whole French Polynesia, in comparison with 5% of Marquesan endemics today. Apart from the high number of introduced species, the explanation is linked to the remoteness of most of these endemics which are often restricted to mountain ridges or steep slopes and which necessitate long journeys to collect them. Some of them are also becoming increasingly rare due to new threats (plants and animals invasive species) combined with some overexploitation (*Rauvolfia* and *Alyxia* bark, sandalwood). Cosmetopoeia appears to be in great evolution; with traditional practices being adapted but still thriving.

### Comparison with pharmacopoeia

Marquesan cosmetopoeia and pharmacopoeia [16] have many similarities due to the lack of a clear boundary in the Marquesan traditional concepts of health and well being. The frameworks of recipes and ways of preparation are also very close and the importance of prevention is also to be noticed. The number of plant species involved is nearly identical (pharmacopoeia 77, cosmetopoeia 79) but their biogeographical status show some differences with slightly more endemic species in cosmetopoeia (4 versus 3) and a switch between Polynesian and modern introductions. Indeed, modern introductions are clearly preferred in cosmetopoeia (44% versus 32%) in comparison with Polynesian introductions, which are more numerous in pharmacopoeia (42% versus 28%). That indicates a higher fidelity of pharmacopoeia to traditional medicinal plants and higher innovation potential with cosmetic plants.

## Conclusions

This study described the cosmetic flora of Marquesas Islands together with its uses. This field survey focusing on cosmetopoeia is the first conducted in French Polynesia, and, at the best of our knowledge, in the Pacific. Indeed, pharmacopoeia was pretty much investigated in the Pacific [29] and even in the Marquesas archipelago [16].

The current Marquesan cosmetopoeia is composed of at least 79 cosmetic plant species of which 28% are native and 72% are introduced and more or less naturalised. Most of these plant species are not threatened by overexploitation excepting three endemics: *Rauvolfia nukuhivensis* (bark), *Alyxia stellata* var. *marquesensis* (bark) and *Santalum insulare* var. *marchionense* - Marquesan sandalwood (heartwood). However, these species and most of the native ones face the major problem of invasive species on islands which prevent regeneration and cause the death of mature plants: rats eating seeds, herbivores grazing seedlings and eating bark, weeds and invasive plants choking juveniles and mature plants. Thus, in order to maintain traditional cosmetic uses, natural populations management of these threatened species combined with plantations to preserve the natural stock are needed. For these reasons, local authorities implemented several years ago ex situ conservation stands of *Rauvolfia nukuhivensis* and *Santalum insulare* on the island of Nuku Hiva. Our study shows that this work is highly relevant and must by pursued and amplified in the context of Marquesan cultural renewal. The main species of the Marquesan cosmetopoeia are a mixture of native species, Polynesian introductions and modern introductions, with the coconut palm (*Cocos nucifera*), from which the coconut oil or monoi is extracted, as the flagship species. This species assemblage indicates the innovation power of Marquesan community in front of new plants and the desire to create new cosmetic products in a traditional framework.

Fragrance appeared an important characteristic of Marquesan beauty concern. Flowers or scented materials are omnipresent in traditional cosmetic preparations (for example in monoi) or directly applied on the hair or on the skin, particularly in scented bouquet [30]. In each interview, there was at least one recipe for perfume or with deodorizing indications. Beauty also involves the softness of skin and the brightness of hair, which is fundamental for Polynesian women proud of their long black hair. The women, except for some specific uses (supercision, tattoo), were the main inheritors and transmitters of traditional cosmetic knowledge to following generations. Unfortunately, nowadays, knowledge transfer is partly broken, with most young Marquesans preferring ready processed products from shops or drugstores instead of preparing traditional recipes and so contributing to the preservation of Marquesan cosmetic knowledge. Thus, simultaneously with the current cultural renewal, it appeared crucial to study and collect this cosmetic knowledge, which is essentially oral.

A close link between cosmetopoeia and pharmacopoeia was demonstrated, some recipes having both cosmetic and medicinal properties. The main plants of the pharmacopoeia were also used in cosmetopoeia, like the well-known Tahitian gardenia *(Gardenia taitensis*)*.*

For a better valorisation of cosmetic plants, phytochemical and specific bioassays studies could be performed. Moreover, ethnobotanical surveys regarding cosmotopoeia should be amplified in order to compare data from different archipelagos of French Polynesia and to document this important cultural wealth, both for its preservation and promotion. We attempted to meet the requirements of the Nagoya Protocol in implementing a Prior Informed Consent (PIC) form dealing also with access and benefit-sharing (ABS), and in working in collaboration with *Académie marquisienne*, an official cultural institution of French Polynesia, aiming at preserving and enriching the Marquesan cultural heritage. Nevermind, proper regulations at the level of the French Polynesian government are needed in order to clarify the local application of access and benefit-sharing concept, especially in the highly promising sector of cosmetics. Indeed, due to lack of local government regulatory framework and especially of the implementing decrees of the French Polynesian law 2012–5 from the 25 January 2012 on ABS, each study based on traditional knowledge has to develop its own ethical methods and to identify the proper representatives of traditional knowledge holders.

## Abbreviations

ABS: Access and benefit-sharing
FL: Fidelity Level
NIE: Number of informants quoting that plant for any use
NIUE: Number of informants quoting one plant species for a particular use
PIC: Prior Informed Consent
